# Predictive value of drug efficacy by M6A modification patterns in rheumatoid arthritis patients

**DOI:** 10.3389/fimmu.2022.940918

**Published:** 2022-08-16

**Authors:** Shan Song, Rong Zhao, Jun Qiao, Jia Liu, Ting Cheng, Sheng-Xiao Zhang, Xiao-Feng Li

**Affiliations:** ^1^ Department of Rheumatology, The Second Hospital of Shanxi Medical University, Taiyuan, Shanxi, China; ^2^ Key Laboratory of Cellular Physiology at Shanxi Medical University, Ministry of Education, Taiyuan, Shanxi, China

**Keywords:** rheumatoid arthritis, epigenetic, m6A RNA modification, immune microenvironment, infliximab

## Abstract

**Background:**

Rheumatoid arthritis is a highly heterogeneous autoimmune disease characterized by unpredictable disease flares and significant differences in therapeutic response to available treatments. One possible reason for poor efficacy is that it cannot be treated accurately due to no optimal stratification for RA patients.

**Objective:**

This study aims to construct an RA classification model by m6A characters and further predict response to medication.

**Methods:**

Twenty m6A regulators were used to construct a random forest diagnosis model, and RNA-seq analysis was employed for external validation. The RNA modification patterns mediated by 20 m6A regulators were systematically evaluated in 1191 RA samples and explored different molecular clusters associated with other immune microenvironment characteristics and biological pathways. Then, we established an m6A score model to quantify the m6A modification patterns. The model was applied to patients at baseline to test the association between m6Ascore and infliximab responsiveness.

**Results:**

The m6A diagnosis model showed good discriminatory ability in distinguishing RA. Patients with RA were classified into three clusters with distinct molecular and cellular signatures. Cluster A displayed strongly activated inflammatory cells and pathways. Specific innate lymphocytes occupied cluster B. Cluster C was mainly enriched in prominent adaptive lymphocytes and NK-mediated cytotoxicity signatures with the highest m6A score. Patients with a low m6Ascore exhibited significantly infliximab therapeutic benefits compared with those with a high m6Ascore (p< 0.05).

**Conclusion:**

Our study is the first to provide a comprehensive analysis of m6A modifications in RA, which provides an innovative patient stratification framework and potentially enables improved therapeutic decisions.

## Introduction

Rheumatoid arthritis (RA) is a highly heterogeneous chronic autoimmune disease that is characterized by joint inflammation (1, 2). Several risk factors are known to be involved in the development of RA, including genetics, female sex, and environmental factors (3). Especially epigenetic regulation (4) is proposed to play an indispensable role in the occurrence and development of RA. Currently, conventional therapies, including conventional DMARDs, biological DMARDs, and targeted DMARDs, have substantially changed the course of RA (5). However, individual responses vary widely to treatment; 6%–21% of patients are refractory to multiple therapies, defined as refractory rheumatoid arthritis (6). Therefore, biomarkers are urgently needed to stratify patients and assess the benefits of from specific drug or class of drugs.

Epigenetics is a branch of genetics that refers to heritable chromosomal changes without nucleotide sequence alterations, including histone modification, DNA methylation, and RNA modification. As the third layer of epigenetics, more than 170 different types of RNA modifications, including N6-methyladenosine (m6A), 5-methylcytosine (m^5^C), and N1-methyladenosine (m^1^A), have been described (7). M^6^A is one of the most dominant RNA modifications in RNA. Like DNA or histone modification, m6A modification is a dynamic and reversible process in mammalian cells controlled by enzymes such as methyltransferases, demethylases, and binding proteins (8). Methyltransferase promotes m6A methylation modification to RNA, and demethylase removes the m6A-methylated group from RNA. RNA-binding proteins bind to the m6A methylation site in RNA to regulate mRNA metabolism and function (9). An in-depth investigation of these regulators would help uncover the role and mechanisms of m6A in gene posttranscriptional regulation (10, 11). Further, accumulating evidence indicated that not only m6A but also m6A regulators were correlated with disorders of multiple biological processes such as the occurrence of tumor (12), disturbance of immunomodulatory function (13), and systemic lupus erythematosus (14). In addition, m6A modification has been implicated in T-cell differentiation, homeostasis, and response to HIV infection (14). Considering the vital role of m6A modification in the immune response and immune cells, m6A may be involved in the etiology of RA. Studying epigenetic factors and mechanisms related to RA progression and treatment response is increasingly significant (15, 16).

However, the epigenetic modulation of single m6A regulators and the overall m6A modification characteristics in RA have not been fully understood. This study aims to revolve the patterns of m6A modifications of RA by performing a comprehensive analysis of the publicly available transcriptome datasets.

## Methods

### Overview of data processing and analysis

A total of 12 RA patients and five healthy controls (HC) were recruited for this study from the Second Hospital of Shanxi Medical University in August 2021. All the patients met the 2010 American College of Rheumatology (ACR)/European League Against Rheumatism (EULAR) classification criteria for RA (17). To evaluate the relationship between m6A and response to treatment, genome-scale data about infliximab therapy response were also recorded in our study, which included patients’ response to anti-TNF evaluated by Disease Activity Score-28(DAS28).

### Preparation of peripheral blood samples and isolation of RNA

Peripheral blood samples (5 ml) were collected from each patient and control subject into EDTA-2 K-containing tubes. According to the manufacturer’s protocol, fresh PBMCs from each donor blood were isolated by Ficoll-Hypaque (Beijing Solarbio Science & Technology Co., Ltd.) density-gradient centrifugation for 20 min at room temperature. Total RNA was isolated from freshly obtained PBMCs. Before RNA sequencing, the quality of RNA was assessed on the Agilent Bioanalyzer 2100 system. A 1.5-μg RNA sample was taken for RNA sequencing. The library preparations were sequenced on Illumina. Removing reads containing adapter, containing ploy-N, and low-quality reads containing >50% bases with qualities of ≤20 from raw data, clean reads were obtained.

### RNA-seq expression analysis

Reference genome and gene model annotation files were downloaded directly from the Genome website, and paired clean reads were aligned to the reference genome using Hisat2 v2.0.5. Reads mapped to each gene were calculated using featureCounts v1.5.0-p3. Then the FPKM for each gene was calculated.

### The random forest model could distinguish between RA and HCs

Samples from the GEO cohort were randomly divided into the training set (935 samples, 70%) and the testing set I (400 samples, 30%). During training, to avoid the overfitting problem caused by random oversampling, the SMOTE sampling method was adopted to repeatedly sample the healthy controls, which had fewer samples, and thereby balance the number of samples in the RA and HCs. The R package “pROC” was used to evaluate the visualization of the receiver operating characteristic curve (ROC) to calculate the area under the curve (AUC). The sequencing data were employed for external validation.

### Unsupervised clustering for 20 m6A regulators

Twenty-one acknowledged m6A regulator genes were referred (10, 18, 19). Only 20 regulators were stably expressed and used to identify distinct m6A methylation modification patterns. These 20 m6A regulators included eight methyltransferases (METTL3, METTL14, RBM15, RBM15B, WTAP, VIRMA/KIAA1429, CBLL1, ZC3H13), two demethylases (ALKBH5, FTO), and 10 RNA-binding proteins (YTHDC1, YTHDC2, YTHDF1, YTHDF2, YTHDF3, HNRNPA2B1, HNRNPC, FMR1, LRPPRC, ELAVL1). Unsupervised clustering analysis was applied to identify different m6A modification patterns based on these 20 m6A regulators by the “ConsensusClusterPlus” package. The optimal and stable numbers of clusters were selected according to cophenetic, dispersion, and silhouette coefficients.

### Annotating immunocyte and function

To characterize the biological features between different m6A modification patterns, we utilized single-sample gene set enrichment analysis (ssGSEA) to estimate the population of specific infiltrating immunocytes and the activity of immune reactions. The gene sets marking each infiltrating immunocyte type were obtained from the previous study17, and the RA-related pathways were obtained from the MSigDB database. The enrichment scores defined by ssGSEA analysis represent the degree of each immunocyte abundance and immune reaction activity in each sample, compared to three distinct modification patterns by the Wilcoxon test.

### Identifying m6A modification phenotype-related DEGs

M6A-related differentially expressed genes (DEGs) in different m6A phenotypes were identified by the “Limma” R package, in which *P-values*< 0.05 were set as cutoff criteria for the DEGs. GO and KEGG enrichment analyses were applied to analyze the biological significance of DEGs. *P*< 0.05 was considered statistically significant, and visualization of results was conducted by R package ‘ggplot2’ and ‘GOplot’.

### Construction of an m6A gene signature

To quantify m6A modification patterns in individual RA patients, we constructed a set of the m6A gene signature (m6Ascore) by using principal component analysis (PCA) algorithms. The differentially expressed genes (DEGs) obtained from three clusters in the previous step were intersected to get shared DEGs between three m6A clusters. Pearson correlations were performed to obtain positive or negative correlation signature genes for co-expressed DEGs. The signature genes were further selected for features by the “Boruta” R package. We then conducted PCA based on the final determining genes. Principal components from the two groups of signature genes were separately extracted and served as the final signature score by subtraction. This approach concentrates the score on the set with highly correlated or anticorrelated gene blocks while down-weighting the gene contributions that are not tracked with other set members. We then define the m6Ascore by adopting a formula like previous studies (18, 20, 21).


Gene.score=∑pca1i−∑pca1j

where i is the signature score of clusters that have a positive coefficient, and j is the expression of genes that have a negative coefficient.

## Results

### Transcriptional alterations of 20 m6A regulators

**Figure 1:**
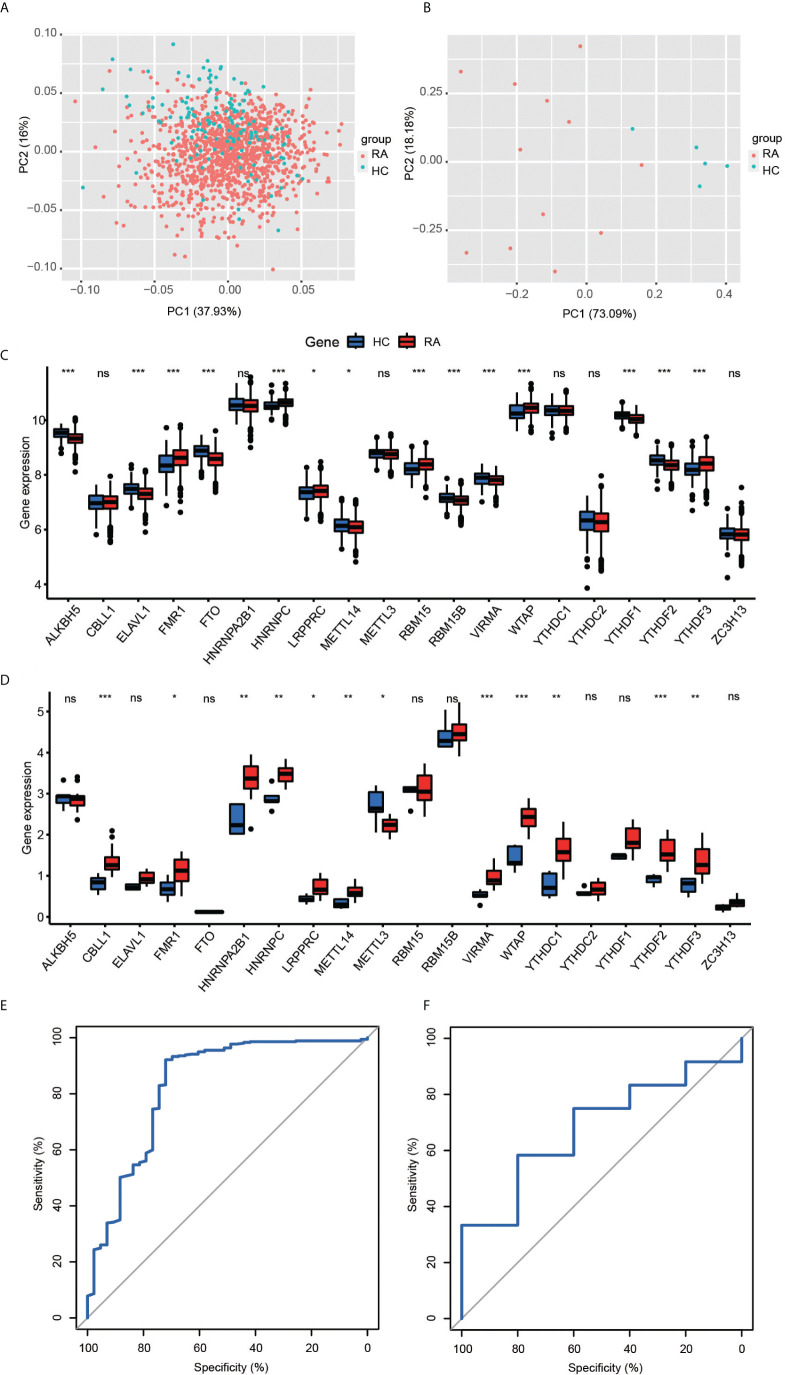
**(A, B)** Principal component analysis for the expression profiles of 20 m6A regulators could roughly distinguish RA from HC samples in the GEO cohort **(A)** and validation cohort **(B)**. **(C, D)** The box plot demonstrated the 20 m6A regulators between healthy and RA patients in the GEO cohort **(C)** and clinical validation cohort **(D)** (*P < 0.05, **P < 0.01, ***P < 0.001, ns P >=0.05). **(E, F)** ROC curve of the 20 specifically expressed m6a regulators in RA samples. The 20-M6A feature random forest model predicted the testing cohort (E, AUC = 0.83) and validation cohort (F, AUC = 0.68).

A total of 1,203 RA and 149 healthy controls (HCs) from six cohorts were included in this study (**Supplementary Table 1**). PCA was used to visualize variation in correcting for batch effects (**Supplementary Figure 1**). We noticed a very close association among methyltransferases in the 20-m6A regulator protein–protein interaction (PPI) network, which usually function as a complex (**Supplementary Figure 2**). Subsequently, we explored the different expressions of 20 m6A regulators between RA and healthy controls in a GEO public cohort (**Figures 1A–C**). Five regulators were observed to have consistently different expressions in 12 RA patients and five HC participants from the Second Hospital of Shanxi Medical University ward and health examination center again (**Figure 1D**): FMR1, HNRNPC, LRPPRC, WTAP, YTHDF3. To examine the ability of the 20 m6A regulators to distinguish between RA and HCs, we conducted a diagnosis model by the random forest (RF) algorithm. The area under the curve (AUC) value was 0.83 (**Figure 1E**). External validation was performed for the diagnostic efficacy of the m6A-RF model (**Figure 1F**). Actually, this diagnostic model is not highly efficient in the validation cohort because of the insufficient sample size, but it also partly explains the role of m6A in the occurrence and development of RA.

### Mediation of m6A RNA methylation modification patterns by 20 regulators

**Figure 2:**
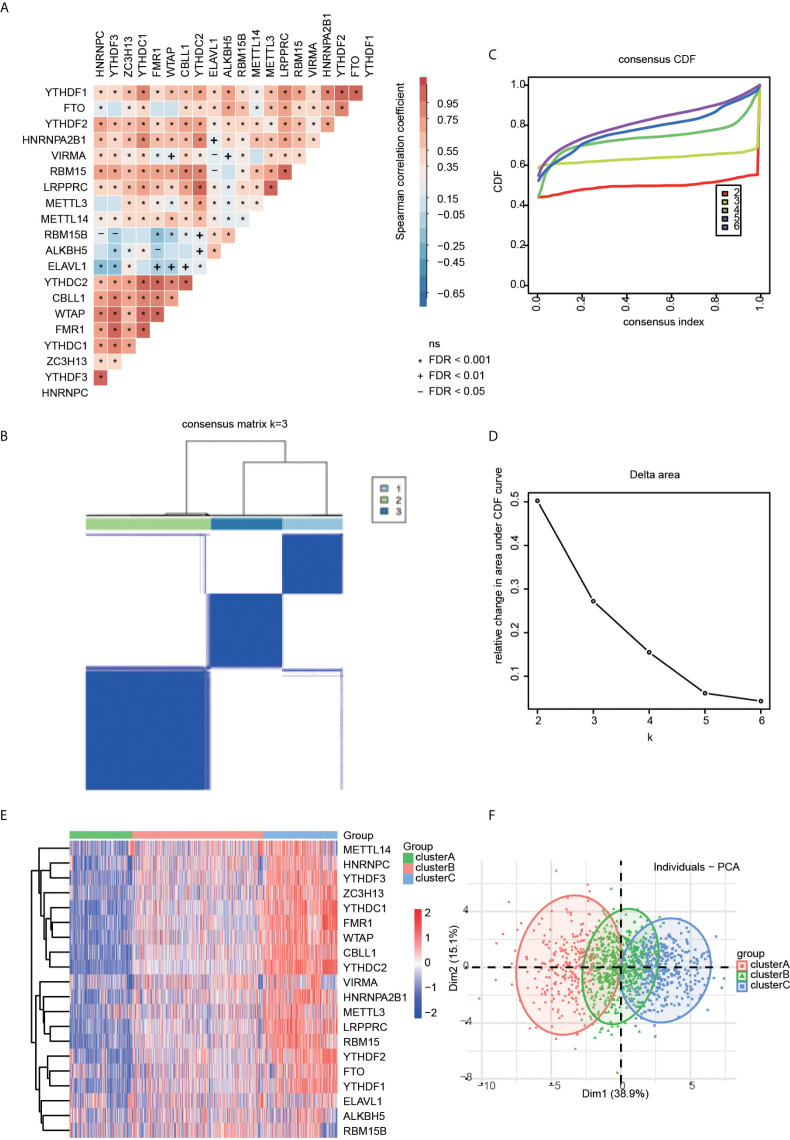
**(A)** Correlation heatmap for all 20 m6a regulators in RA patients. Some regulators were negatively related, represented in blue, and others were positively correlated, represented in red. The darker the color, the higher the correlation. **(B)** The consensus cluster matrix for k = 3 shows three major clusters. **(C)** Consensus clustering cumulative distribution function (CDF) for k = 2–6, which can completely describe the probability distribution of a real random variable. **(D)** The relative change of CDF Delta area curve for k = 2–6. **(E)** The distribution of 20 m6A RNA methylation regulators among three clusters. **(F)** Visualization of the clustering results through a scheme based on principal component analysis (PCA).

To investigate transcriptome relationships, we calculated pairwise correlations among the expressions of the 20 m6A regulators. We found that positive correlations were more frequent than negative correlations (**Figure 2A**). Next, based on the expression profiles of the 20 selected m6A regulators, we utilized consensus clustering analysis to stratify patients to different m6A modification patterns (**Figures 2B–D**). Accordingly, we determined that the matrix heatmap retained sharp and clear sides when k = 3, which indicated there were three distinct m6A modification pattern clusters, including 279 cases in cluster A, 581 cases in cluster B, and 331 cases in cluster C (**Figures 2E, F**). We termed these clusters as m6A cluster A, m6A cluster B, and m6A cluster C.

### Immune landscape characteristics in m6A modification patterns

**Figure 3:**
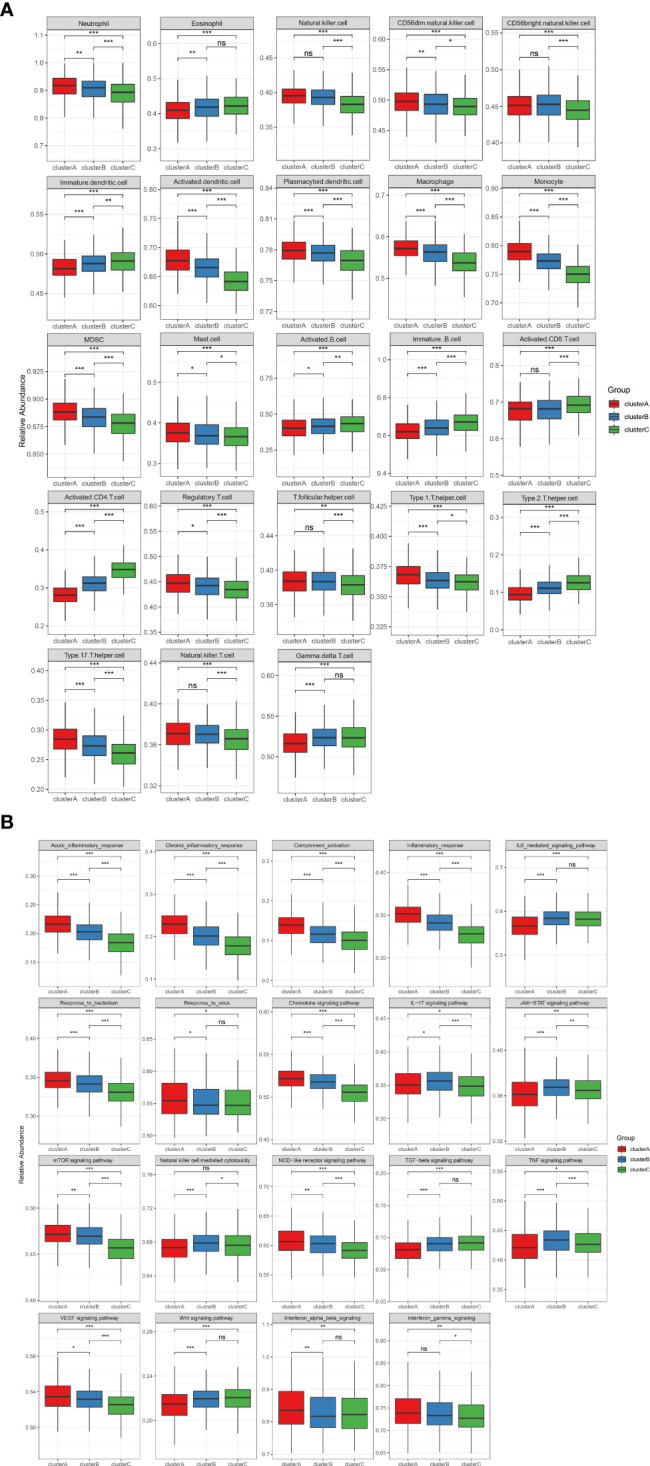
**(A)** The abundance of infiltrating immunocytes cells in the three m6A clusters. The upper and lower ends of the boxes represent the interquartile range of values. The lines in the boxes represent the median value, and the black dots show the outliers. The asterisks represent the statistical p-value (*P < 0.05, **P < 0.01, ***P < 0.001), ns P>=0.05. **(B)** Some rheumatoid arthritis-related immune reaction gene sets showed the activity differences in the three m6A clusters (*P < 0.05, **P < 0.01, ***P < 0.001), ns P>=0.05.

To identify the immune microenvironment characteristics underlying three distinct m6A modification patterns, we compared the enrichment scores of RA-related pathways and immune cell infiltration (**Supplementary Tables 2, 3**) among the RNA modification patterns. We found inflammatory cell infiltrates, including neutrophils, monocytes, and T helper type 17 (Th17) in cluster A, with the enormous imbalance between T helper type 1 and T helper type 1 (Th1/Th2) at the same time (**Figure 3A**). Patients in cluster C were rich in adaptive immune-related cells such as activated CD4+T cells, activated CD8+T cells, and activated B cells (**Figure 3A**). In cluster C, Th2 and eosinophil abundant infiltration was considered a protective factor by counteracting the development of arthritis and preventing bone loss20. Cluster B was modestly activated in most inflammatory and immune cells. Nevertheless, cluster B displayed more activation of natural killer cells (NK), natural killer T cells (NKT), gamma delta T cells (γδT), and CD56 bright natural killer cells (CD56bright NK), which are typical innate lymphoid cells. In parallel with this, the RA-related biological process was differentially activated in the three subgroups (**Figure 3B**). Cluster A showed strong enrichment for most inflammatory pathways, including acute and chronic inflammation, response to bacterium and virus, complement activation, and chemokine. Therefore, cluster A can be referred to as the highest inflammatory phenotype. In contrast, the abovementioned inflammatory pathways were remarkably less expressed in cluster C than in clusters A and B, and we assume cluster C as an adaptive lymphocyte-rich phenotype. Cluster B was enriched in natural killer cell-mediated cytotoxicity, IL-17 signaling pathway, JAK-STAT signaling pathway, and TNF signaling pathway. They were identified as an innate lymphocyte-rich phenotype.

### Clusters are not influenced by disease activity

**Figure 4:**
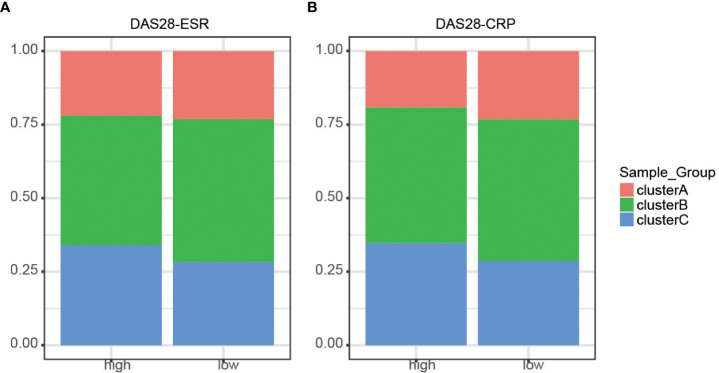
Distribution of disease activity scores between clusters. **(A)** DAS28-ESR showed no discrepancies identified in the three m6A clusters (P > 0.05). **(B)** DAS28-CRP showed no discrepancies identified in the three m6A clusters (P > 0.05).

To determine whether the molecular cluster has an association with clinical features, we investigated the distribution of the three clusters according to disease activity (**Figure 4** and **Supplementary Table 4**). Disease Activity Score (DAS)28 - erythrocyte sedimentation rate (DAS28-ESR) and DAS28-C-reactive protein (DAS28-CRP) larger than 5.1 were regarded as high disease activity. All three clusters existed independently of disease status. Therefore, the three clusters were similar and clinically indistinguishable by DAS28-ESR/DAS28-CRP parameters.

### M6A phenotype-related DEGs

**Figure 5:**
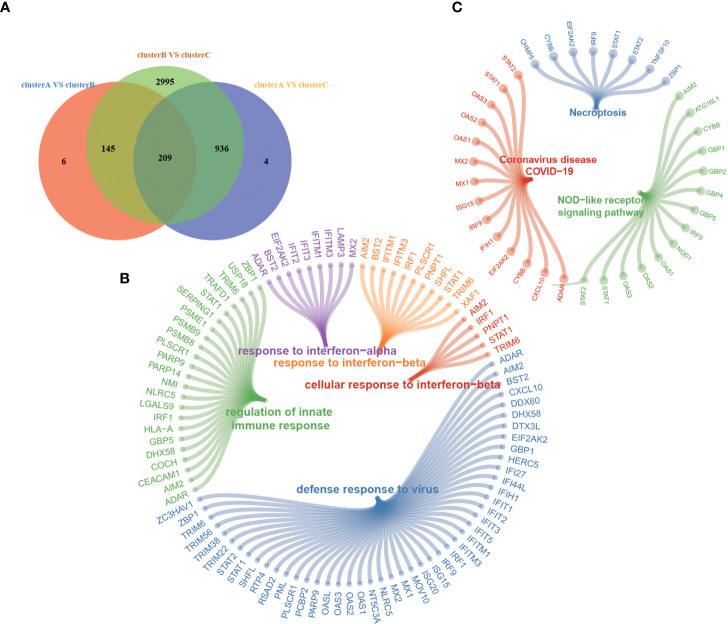
**(A)** Venn diagram shows 209 overlapping differentially expressed genes (DEGs) among the three m6A modification patterns. **(B, C)** Functional annotation for 209 m6A phenotype-related DEGs by GO **(B)** and KEGG **(C)** enrichment analyses. Each node represents one pathway or gene.

Although RA patients were classified into three m6A modification phenotypes based on 20 m6A regulators, the underlying genetic changes and expression perturbations within these phenotypes remain unclear. To further examine potential m6A-related transcriptional expression changes in three patterns, we identified 209 overlapping DEGs using an empirical Bayesian algorithm and performed an enrichment analysis (**Figure 5A**). GO and KEGG pathway enrichment analyses were conducted to explore the functional characteristics of the DEGs (**Figures 5B, C**). The GO and KEGG analysis results showed that the DEGs were significantly enriched in “defense response to virus”, “response to interferon-beta”, “response to interferon-alpha”, “regulation of innate immune response”, “NOD-like receptor signaling pathway”, “Coronavirus disease-COVID-19”, and so on. We performed an unsupervised consensus clustering analysis based on the 209 RNA phenotype-related DEGs to further validate this differential regulation. Consistent with the clustering grouping of m6A modification patterns, we obtained three stable transcriptomic phenotypes named m6A gene clusters A–C (**Supplementary Figure 3**), consistent with the expected results.

### M6A score model in the value of drug response prediction

**Figure 6:**
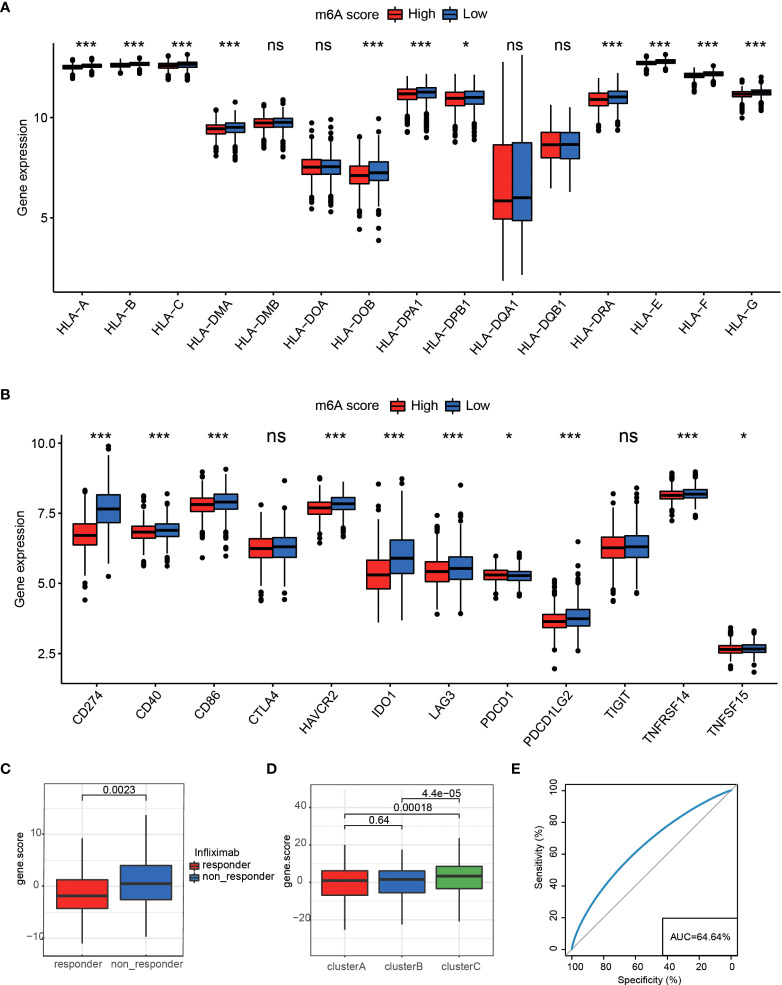
**(A)** The expression differences of HLA between two different groups *P < 0.05, ***P < 0.001, ns P>=0.05. **(B)** Differences in 12 critical immune checkpoints between high m6Ascore and low m6Ascore groups *P < 0.05, ***P < 0.001, ns P>=0.05. **(C)** M6Ascore in the infliximab treatment responder and non-responder patients. **(D)** M6Ascore in three m6A clusters (Wilcoxon test). **(E)** ROC curve of the m6Ascore in RA responder and non-responder samples, suggesting that the m6Ascore feature could predict patients’ response levels.

We applied the m6A score model to accurately evaluate the m6A modification pattern of individual patients with RA. Patients were divided into high or low m6A score groups using the median m6A score as the cutoff. In the two m6Ascore groups, almost all HLA gene expressions showed prominent differences, and that in m6Ascore-low was significantly higher than that of m6A score-high (**Figure 6A**). The m6Ascore-high group observed a significantly lower expression of 12 critical immune checkpoints than that in the m6Ascore-low group, like tumor necrosis factor superfamily15, tumor necrosis factor receptor superfamily14, CTLA4, and CD86 (**Figure 6B**). The above analysis indicated that m6Ascore might be closely connected with immunotherapy. To further understand the effects of m6Ascore on predicting drug response, we selected two independent groups, 154 RA patients treated with infliximab (**Supplementary Table 5**) and 92 RA patients treated with rituximab (**Supplementary Table 6**). We tested the differences in m6Ascore between infliximab responders and non-responders and found that responders had a lower m6Ascore, while patients showed a high m6Ascore with poor clinical efficacy of infliximab therapy (**Figure 6C**). The above analysis was in accordance with the expected results that the lower m6A score group, which has higher immune checkpoints TNFSF15 and TNFRSF14, may have a better anti-TNF (like infliximab) treatment effect. In cluster C, a higher m6A score was observed, which suggested that there may be a lower infliximab therapeutic response. Clusters A and B have lower m6A scores, and there may be higher infliximab therapeutic responses (**Figures 6D, E**). Nevertheless, the rituximab cohort study did not observe significant treatment differences between the two m6Ascore groups (**Supplementary Figure 4**).

## Discussion

Heterogeneity within RA remains poorly characterized and understood; the possible reason might be that the existing classification criteria for RA do not clearly explain the heterogeneous clinical response to the different treatments. Some studies tried to search for new stratification and classification in rheumatoid arthritis. The type I interferon signature expression at baseline could predict clinical outcomes upon TNFα blockade treatment (22). Frances et al. stratified RA as B-cell-poor and B-cell-rich patients. They found that patients with a low or absent B-cell lineage expression signature in synovial tissue tocilizumab are more effective than rituximab (23). To explore the contribution of m6A subtype classification to the heterogeneity of RA, we developed an m6Ascore model to quantify the m6A modification patterns of individual RA patients. We assessed its potential predictive value in infliximab therapy.

Along with research going deep, several studies have partially unraveled the relationship between m6A modification and RA. RNA modifications play an indispensable role in autoimmune regulation through an interaction with diverse m6A regulators (24, 25). Specific deletion of m6A methyltransferases causes severe autoimmune diseases (13). Luo et al. confirmed that the expressions of peripheral blood ALKBH5, FTO, and YTHDF2 were associated with disease activity and inflammatory response (26). METTL3 serves as a potential biomarker for the diagnosis of RA due to its significant inhibition of the inflammatory response of macrophages (27). Therefore, identifying the roles of m6A modification patterns in RA will help understand the mechanisms of m6A in RA, providing insights into the prediction of the efficacy of immunotherapy strategies.

First of all, we identified the distinct expression of the m6A regulator pattern of RA patients. The classifier based on 20 m6A regulators could distinguish RA and healthy individuals, reaffirming the critical role of m6A regulators in RA. Then, we identified three distinct RNA modification patterns correlated with different immune phenotypes. Considering that stratification methods are not clinically practical enough, we established a scoring system to quantify the m6A modification patterns of individuals and accurately reflected the m6A regulator modification pattern in RA. We found that the m6A score not only played a non-negligible role in shaping different m6A methylation modification patterns but also might define clinically meaningful subgroups of RA patients with distinct responses to therapeutic agents. Patients sensitive to infliximab therapy were significantly related to a lower m6A score. Interestingly, the predictive value of the m6A score in rituximab-treated cohorts has not been observed. Previous studies found inherent molecular signatures in RA patients, independent of disease severity and which could not be entirely normalized with current symptomatic treatments (28). In addition, the feature is associated with resistance to drug treatments, which are primarily explained by the imbalance of immune cell subsets (28). In our results, RA was classified into three clusters, (A) highest inflammatory phenotype, (B) innate lymphocyte-rich phenotype, and (C) adaptive lymphocyte-rich phenotype, and there was no differential distribution of disease activity scores between clusters. Cluster A showed strong inflammatory features including a left shift in neutrophil- and monocyte-activated complement pathways. Tasaki et al. found that transcriptional changes induced by infliximab treatments mainly occurred in genes expressed in neutrophils (28). Furthermore, one study also reported good responders for infliximab-exhibited mobilization of neutrophils and monocytes, whereas poor responders showed a high expression of activated B-cell genes (29). In contrast, cluster C showed the lowest levels in nearly all inflammatory cells and pathways and had abundant activated B-cell and immature B-cell infiltration, which could be important reasons for cluster C patients with an unfavorable response to infliximab. The highest m6A score implies a worse response to infliximab in our results. Additionally, cluster A has been significantly activated in the mTOR signaling pathway, reported in several chronic inflammatory diseases. The inhibition of mTOR has shown moderate efficacy in reducing joint inflammation (30). Cluster B is an interesting subgroup, which was modestly activated with neutrophils, monocytes, and B cells. However, its m6A score is comparable to cluster A. Except for neutrophils, Tasaki et al. also found that the second most informative cell type in the infliximab response model was NK (28). Infliximab was observed to bind firmly to CD14^dim^ monocytes, granulocytes, and NK cells, hardly binding to CD8+ T cells or B cells, which might be related to expressing higher levels of membrane TNF (mTNF) in RA patients (31). Infliximab induces potent anti-inflammatory responses through TNF (32). In this context, the response to infliximab of cluster BC may be related to the highest activation with innate lymphocytes represented by NK.

M6A-related gene signatures were used for pathway enrichment analysis and found to have significant differences in innate immune responses among the three clusters, consistent with characteristics of immune cell infiltration. Plenty of studies have revealed nucleotide-binding and oligomerization domain (NOD)-like receptors (NLRs) to play essential roles in autoimmune diseases including RA (33, 34). In addition, increasing studies have reported a role for type I IFNs (interferons) in the pathogenesis of different subsets of RA patients, indicating that IFN-α/βactivity may have essential clinical utility in predicting response to tumor necrosis factor antagonists (35–37). Chen et al. also found that the expression of m6A phenotype-related hub genes might predict a therapeutic response to anti-TNF therapy in inflammatory bowel disease (38). Previous studies have demonstrated that m6A involves the progression of the inflammatory response by affecting TNF-α degradation and regulation. The knockdown of METTL14 led to the inhibition of the TNF-α-induced cell senescence (39), endothelial inflammation, and atherosclerosis development (40). Tong et al. have confirmed that METTL3-deficient macrophages exhibited reduced TNF-α production upon LPS stimulation *in vitro (* 41). YTHDF2 knockdown significantly increased the LPS-induced IL-6, TNF-α, IL-1β, and IL-12 expression (42), accompanied by increased TNF receptor superfamily member 1b (TNFRSF1b) mRNA (43).

Our study is the first to systematically analyze the relationship between m6A regulators and rheumatoid arthritis. We identified three distinct m6A methylation modification patterns and constructed a scoring model which demonstrated clinical utility for the m6A score as a biomarker in the prediction of arthritis infliximab therapy, which are likely to be useful in future studies exploring m6A epigenetic modification in RA and potentially enabling improved therapeutic decisions and more reliable prediction of response to therapy. Nevertheless, it can hardly be denied that this study had some limitations. This study is based on bioinformatics analysis, and many results theoretically need to be verified by subsequent experiments like m6Aseq, LC-MS, and MeRIP seq.

## Data availability statement

The data presented in the study are deposited in the National Genomics Data Center, China National Center for Bioinformation repository (https://ngdc.cncb.ac.cn), accession number PRJCA010630.

## Ethics statement

The studies involving human participants were reviewed and approved by The Ethics Committee of the Second Hospital of Shanxi Medical University (2016 KY-007). The patients/participants provided their written informed consent to participate in this study.

## Author contributions

Study design and manuscript writing: SS and RZ. Data extraction, quality assessment, analysis, and interpretation of data: SS, RZ, JQ, JL, and TC. Financial support and review: S-XZ, and X-FL. All authors were involved in drafting the article or revising it critically for important intellectual content, and all authors approved the final version to be published. X-FL had full access to all the data in the study and took responsibility for the integrity of the data and the accuracy of the data analysis.

## Funding

The authors disclosed receipt of the following financial support for the research, authorship, and publication of this article: this work was supported by the National Natural Science Foundation of China (No. 82001740).

## Conflict of interest

The authors declare that the research was conducted in the absence of any commercial or financial relationships that could be construed as a potential conflict of interest.

## Publisher’s note

All claims expressed in this article are solely those of the authors and do not necessarily represent those of their affiliated organizations, or those of the publisher, the editors and the reviewers. Any product that may be evaluated in this article, or claim that may be made by its manufacturer, is not guaranteed or endorsed by the publisher.
