# Supplementary material for: Predictive value of drug efficacy by M6A modification patterns in rheumatoid arthritis patients
Source: Front Immunol. 2022 Aug 16;13:940918. doi: 10.3389/fimmu.2022.940918 (PMC9427021; doi:10.3389/fimmu.2022.940918)
Supplement: Supplementary file 1 [file DataSheet_1.docx]

Supplementary Material

##
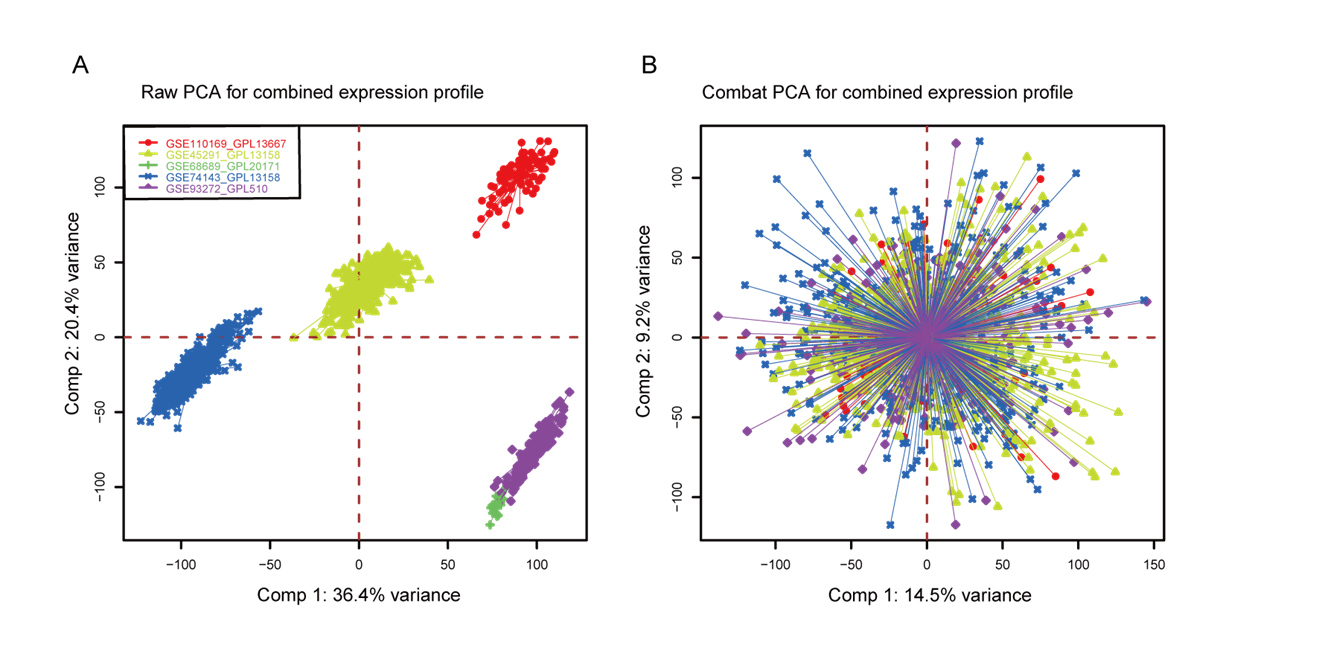
Supplementary Figure 1. (A-B) Batch effects were corrected using the Combat function from the “SVA” R package. Before correction the five groups observed are still evident (A), after batch correction, batch effects appear to be removed (B).


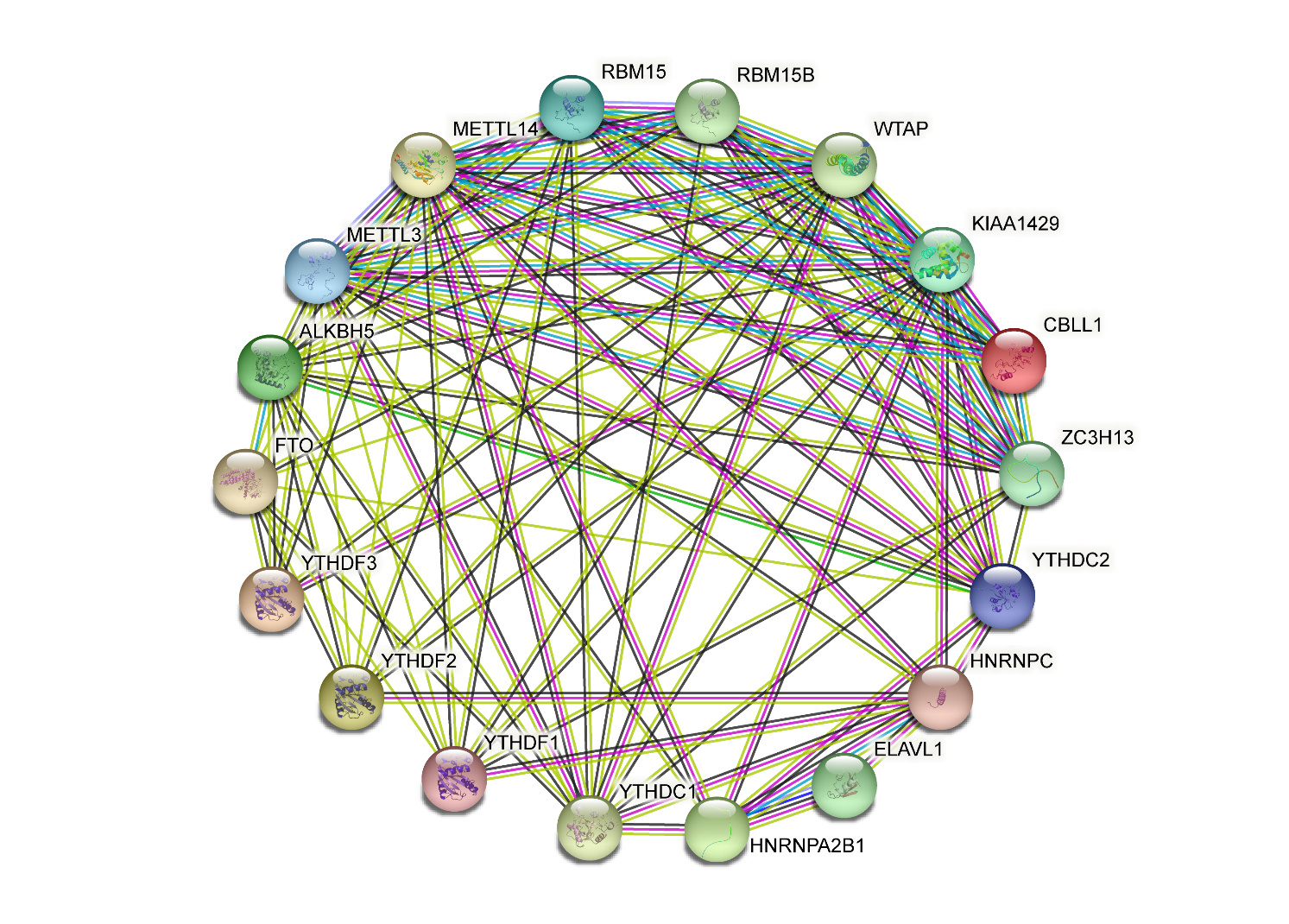


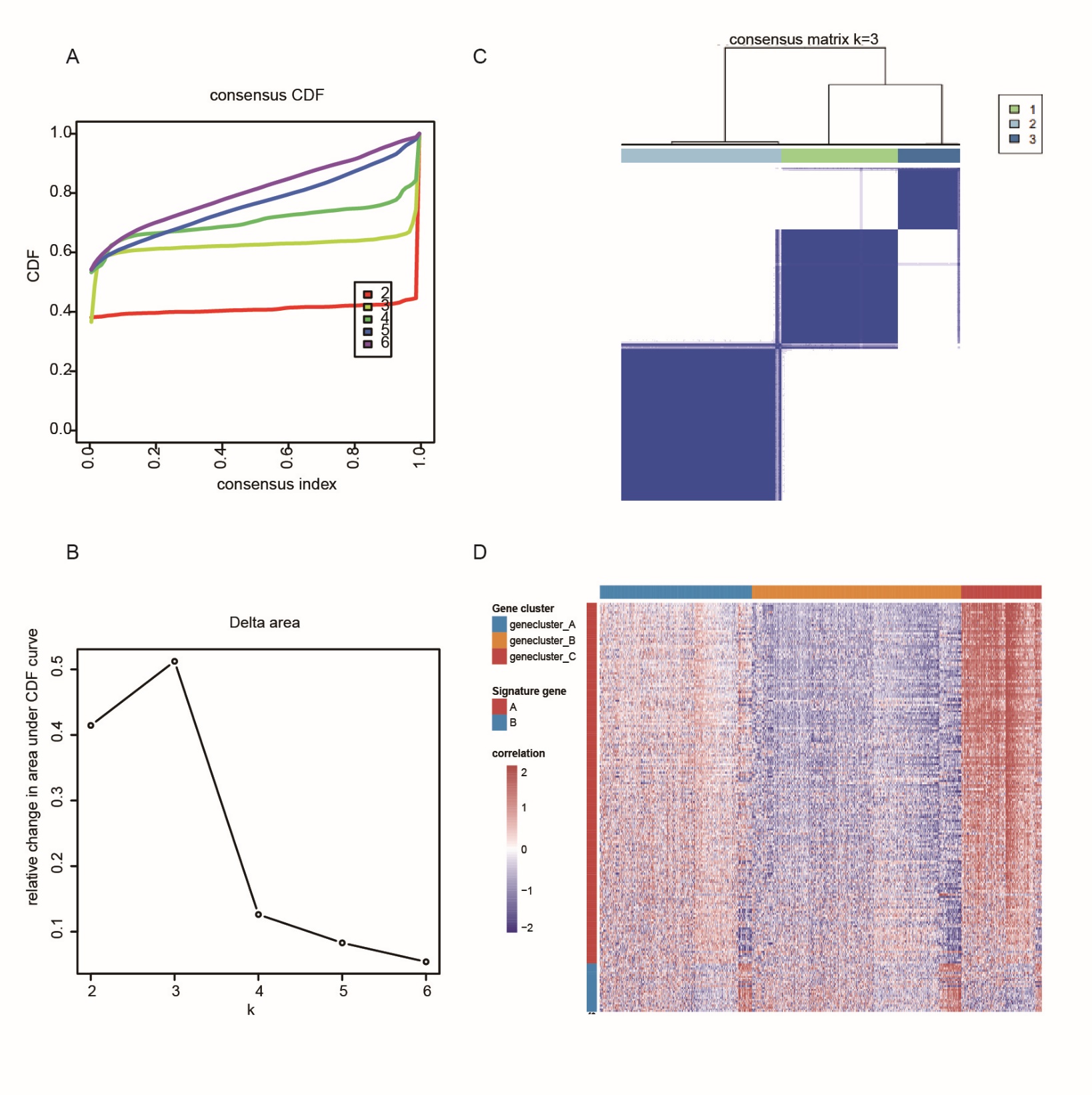
**Supplementary Figure 2.** The interaction network between proteins is coded by 20 m6a regulators. Each node represents a regulator, while each edge represents one protein-protein association.

**Supplementary Figure 3.** (A) Consensus clustering cumulative distribution function (CDF) for k = 2–6, which can completely describe the probability distribution of a real random variable. (B) The relative change of CDF Delta area curve for k = 2–6. (C) The consensus cluster matrix for k = 3 shows three major clusters. (D) Heatmap shows that based on 209 RNA phenotype-related DEGs RA could be stratified into three stable transcriptomic phenotypes.


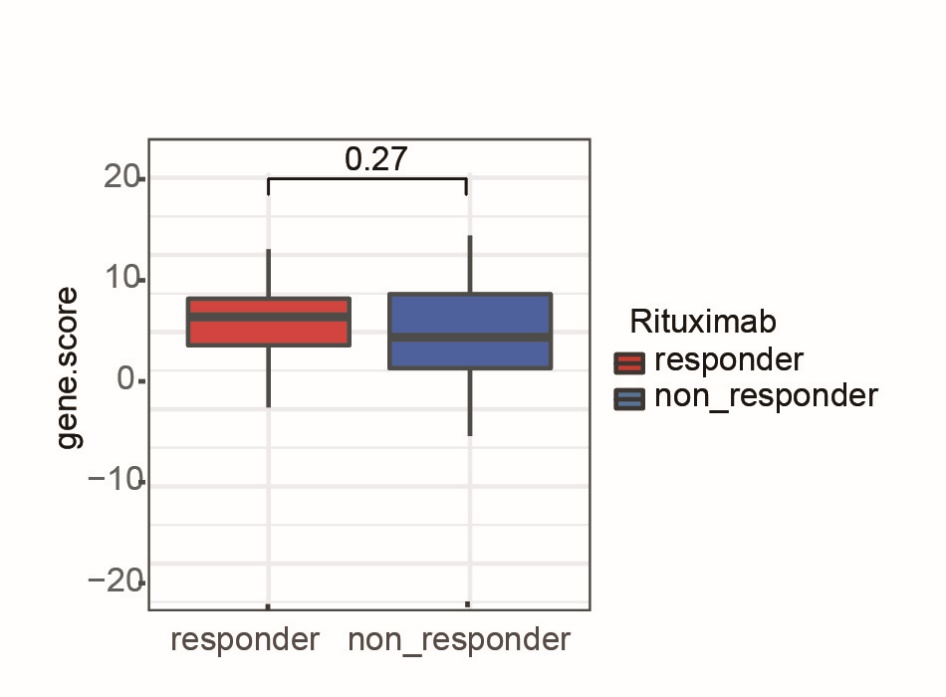


**Supplementary Figure 4.** M6Ascore in the rituximab treatment responder and non-responder patients (Wilcox-test, P=0.27).
